# Creatine supplementation on fatigue related to post-COVID-19 condition—fatigue study: a randomized controlled trial

**DOI:** 10.3389/fnut.2026.1731306

**Published:** 2026-03-25

**Authors:** Maercio Souza Cícero dos Santos, Mariana de Souza Dorna, Adriele Fogaça Costa, Estefânia Aparecida Thomé Franco, Izabele Bassani, Jéssica Geronutti, Letícia Cláudia de Oliveira Antunes, Luís Fernando Pereira Brizola, Carolina Carreira Breda, Caroline Santos Marin, Fernanda Saori Suetake, Yasmin de Oliveira Barros, Paula Schmidt Azevedo, Sergio Alberto Rupp de Paiva, Suzana Erico Tanni, Robson Aparecido Prudente

**Affiliations:** 1 São Paulo State University (UNESP), Medical School, Botucatu, Brazil; 2 Clinical Hospital of the Faculty of Medicine of Botucatu, Botucatu, Brazil

**Keywords:** COVID-19, creatine, exercise, fatigue, post-COVID-19 condition

## Abstract

**Background:**

Post-COVID-19 condition (PCC) is characterized by fatigue, dyspnea, and muscle pain, with treatment including physical exercise and nutritional support. Creatine supplementation under these conditions may increase the total body creatine pool, thereby increasing muscular phosphocreatine availability and improving the energy substrate supply to sustain activity and reduce fatigue. This study investigated the efficacy of creatine supplementation in alleviating fatigue symptoms in patients with PCC, as well as its effects on quality of life, lung function, physical performance, body composition, and muscle strength.

**Methods:**

This randomized, single-blind, placebo-controlled pilot study assessed supplementation with either 6 g or 18 g of creatine per day or 6 g of maltodextrin (placebo) combined with physical activity three times a week over 4 weeks. Participants with PCC who experienced fatigue [score ≥4 on the revised Piper Fatigue Scale (PFS-R)] underwent physical examination; laboratory evaluation; pulmonary function tests; muscle ultrasound; respiratory and peripheral muscle strength assessments; body composition analysis; physical capacity tests; and questionnaires on quality of life, dyspnea, anxiety, and depression before and after the intervention. Difference-in-differences (DID) between these two time points were compared across the intervention groups and the control arm.

**Results:**

Sixty-seven individuals were randomized. Participants were predominantly female (76.6%), with a mean age of 52 ± 12 years, body weight of 82.6 (73.1–93.4) kg, and height of 1.63 ± 0.07 m. A total of 58 completed the protocol: 21 in the 6 g/day group, 19 in the 18 g/day group, and 18 in the placebo arm. When the difference-in-differences (DiD) in the PFS-R before and after the intervention was assessed, a score change of −2.05 was observed in the 6 g/day arm [95% confidence interval (CI): −3.47 to −0.63; *p* = 0.005]. Additionally, this same group showed a significant increase in handgrip strength [mean difference (DiD estimate): 4.40 kgf; 95% CI: 0.27 to 8.52; *p* = 0.037]. Adverse events were minimal, transient, and did not require medical intervention.

**Conclusions:**

Creatine supplementation at 6 g/day was associated with improvements in fatigue and peripheral muscle strength in patients with PCC, with a favorable safety profile.

## 1 Introduction

Since the onset of the COVID-19 pandemic, millions of people have been affected by the post-COVID-19 condition (PCC). According to the World Health Organization (WHO), global estimates indicate that approximately 6% of individuals who have COVID-19 develop PCC (1, 2). Also known as post-COVID syndrome, long-term COVID-19, or simply long COVID, its diagnosis is primarily clinical and is defined by the persistence of symptoms for more than 3 months following severe acute respiratory syndrome coronavirus 2 (SARS-CoV-2) infection, as well as diverse clinical manifestations that may affect multiple organ systems and present with varying degrees of severity (1, 3). More than 200 symptoms extending beyond the respiratory system have been reported, including muscle or joint pain, dyspnea, headache, cognitive impairment, and changes in taste. Among these manifestations, fatigue consistently emerges as one of the most prevalent, disabling, and persistent symptoms, significantly impairing daily functioning, work capacity, and quality of life (2, 4–6).

There is still limited understanding regarding the causes, risk factors, and interventions associated with PCC. However, current studies suggest that PCC shares substantial clinical and pathophysiological similarities with other post-infectious syndromes, particularly myalgic encephalomyelitis/chronic fatigue syndrome (ME/CFS) and post-viral fatigue syndromes (7). Additionally, potential irregularities in cellular bioenergetics have been highlighted in these conditions, particularly those related to mitochondrial dysfunction and impaired adenosine triphosphate (ATP) resynthesis, with emerging evidence also suggesting disturbances in creatine metabolism (8–13). Consequently, current treatment is based on classical interdisciplinary approaches to chronic fatigue management, typically involving physical rehabilitation and nutritional and psychosocial support, with or without pharmacological therapy (14–18).

Early investigations in patients with chronic fatigue syndrome provided preliminary evidence of alterations in muscle creatine turnover, including subtle changes in urinary creatinine excretion, suggesting impairment of the muscular creatine pool and bioenergetic homeostasis (10). Subsequent studies using phosphorus magnetic resonance spectroscopy (31P-MRS) demonstrated a significantly reduced rate of post-exercise phosphocreatine (PCr) resynthesis in these patients compared with sedentary controls, reinforcing the presence of mitochondrial and energetic dysfunction (11). Alterations in cerebral creatine metabolism have also been described, with reduced creatine levels observed in brain regions such as the hippocampus, indicating that bioenergetic impairment may extend beyond skeletal muscle (12).

Based on these findings, the hypothesis that creatine supplementation could attenuate fatigue-related symptoms by increasing the total body creatine pool and PCr availability has been explored. Clinical trials conducted in conditions characterized by chronic fatigue and mitochondrial dysfunction, including fibromyalgia, aging, and untrained elderly populations, have demonstrated increases in intramuscular PCr content, improvements in muscle function, greater resistance to fatigue, and delayed onset of neuromuscular fatigue following creatine supplementation (19–22). More recently, preliminary studies in patients with post-COVID-19 condition suggest beneficial effects of creatine supplementation on energy metabolism and fatigue-related symptoms, supporting its potential therapeutic role in long COVID (23, 24). Nevertheless, despite this established bioenergetic rationale and supportive evidence from related conditions, data specifically evaluating the effects of creatine supplementation on fatigue in patients with post-COVID-19 condition remain limited (23–25).

Thus, the present study was designed to characterize the clinical progression of patients with persistent PCC and to compare the impact of creatine supplementation on symptom evolution. Additionally, the efficacy of this intervention on muscle mass, strength, and physical performance in adults was evaluated. Taken together, these results may inform the development of new therapeutic strategies, contributing to improvements in the quality of life of affected individuals and reducing the burden of COVID-19 on healthcare systems.

In this context, we aimed to evaluate the efficacy of creatine supplementation in alleviating fatigue symptoms in patients with PCC, as well as its effects on quality of life, lung function, physical performance, body composition, and muscle strength.

## 2 Methods

### 2.1 Study design, participants and ethical approval

We conducted a randomized, single-blind, placebo-controlled pilot clinical trial to evaluate the efficacy and safety of creatine supplementation at daily doses of 6 g or 18 g for 28 ± 3 days. Patients attending the multidisciplinary post-COVID-19 outpatient clinic of the Clinical Hospital of Botucatu Medical School (HCFMB/UNESP), São Paulo, Brazil, were invited to participate.

Eligible participants were adults (≥18 years old), male or female, with a documented diagnosis of COVID-19 and fatigue persisting for more than 12 weeks following acute SARS-CoV-2 infection, who were clinically stable at the initial evaluation, who had a negative pregnancy test for women of childbearing potential, and who were able to comply with study visits and procedures. Exclusion criteria included hospitalization or requirement for hospitalization during the study protocol, preexisting severe and uncontrolled organ insufficiency, pulmonary sequelae identified by chest CT and/or pulmonary function tests performed prior to baseline evaluation that were unrelated to COVID-19, use of unregistered nutritional or investigational products within 3 months or within five half-lives before baseline, inability to perform physical exercise/rehabilitation, or psychiatric disorders preventing comprehension of the proposed procedures. The participants provided signed informed consent, and the study was approved by the Research Ethics Committee of the Botucatu Medical School (CAAE: 65312822.8.0000.5411).

Participants were randomized at a 1:1:1 ratio into three groups: (1) creatine 6 g/day, (2) creatine 18 g/day, and (3) placebo (maltodextrin 6 g/day). The randomization list was generated by an independent coordinator and stratified by age (>40 years) and sex. Randomization was performed through a centralized online system, with block randomization implemented in REDCap and integrated with the stratification factors. The treatment assignment was revealed only after all participant enrollment information had been entered into the system. Participants were blinded to their group assignment (single-blind design). All baseline and end-of-treatment evaluations were conducted by an independent investigator who was unaware of the assigned supplementation. Creatine supplementation was provided as creatine monohydrate, packaged identically across groups. Participants were instructed to ingest the supplement once daily, diluted in water, preferably at the same time each day.

All participants underwent the same standardized physical rehabilitation program, consisting of 3 weekly sessions (two in-person and one remote), supervised by a qualified cardiorespiratory physiotherapist. The program included aerobic exercise, strength training, flexibility, balance, neuromuscular control, and respiratory exercises. Given the reduced exercise tolerance commonly observed in post-COVID-19 condition, exercise intensity was individualized based on baseline assessment. Aerobic training was prescribed at moderate to high intensity (60%−70%) using the Karvonen formula based on heart rate reserve, with maximal heart rate estimated as 220—age (26). Exercise modalities included walking, treadmill, or cycle ergometer training. Resistance training was performed at approximately 65% of one-repetition maximum using elastic bands, dumbbells, or body weight. Inspiratory muscle training was conducted with a threshold device at ≥30% of maximal inspiratory pressure, in three sets of 15–30 breaths. Balance training, breathing exercises, and bronchial hygiene techniques were included when clinically indicated. Exercise sessions were progressively adjusted according to individual tolerance, whereas supplement doses remained unchanged. Adherence to the exercise program was defined as completion of at least 60% of the prescribed sessions and was verified by attendance records for in-person sessions and self-reported participation in remote sessions.

At the baseline visit (D1), participants diagnosed with PCC and fatigue by a qualified physician were evaluated and subsequently randomized into one of the three study groups. They received the assigned supplement in identical packaging, were given all instructions regarding supplement use, and had their rehabilitation sessions scheduled. The first week of supplementation was provided after the initial assessment, whereas subsequent weekly supplies were delivered during the in-person physiotherapy sessions, following verification of adherence to the previous week by counting and identifying all returned packages. Missing supplementation for at least 60% of the days in the week (4 days) was considered a major protocol deviation. Prior to the start of the first weekly rehabilitation session, the participants were also asked about their general health status, supplement usage and administration times, occurrence and intensity of any adverse events, unplanned use of healthcare resources, and any changes in concomitant medications.

At the end-of-treatment (EoT) visit, participants were reassessed following the initial evaluation protocol, including the presence of adverse events, concomitant medications, unplanned medical visits, and hospitalizations.

An independent efficacy and safety committee conducted biweekly reviews of clinical and safety data, including cumulative reports and emergency analyses. The committee monitored data quality and, although empowered to recommend suspension or termination of the study, this was not necessary owing to the absence of serious adverse events, thereby ensuring participant safety.

### 2.2 Assessments and procedures of the study

Fatigue, defined *a priori* as the primary outcome, was assessed using the Piper Fatigue Scale–Revised (PFS-R), validated for use in the Brazilian population, with fatigue defined as a score >4 (27–29). Secondary outcomes included quality of life, psychological symptoms, dyspnea, pulmonary function, body composition, muscle morphology, exercise capacity, muscle strength, and laboratory parameters. Quality of life was evaluated using the Brazilian Portuguese version of the Saint George's Respiratory Questionnaire (SGRQ) (30). Symptoms of anxiety and depression were assessed with the Hospital Anxiety and Depression Scale (HADS) (31). Dyspnea was measured via the Modified Medical Research Council (mMRC) scale and the Baseline Dyspnea Index (BDI), both of which have been validated for Brazilian Portuguese and cultural contexts (32, 33).

A complete pulmonary function test was performed via computerized systems (CareFusion Germany 234 GmbH, Hochberg, 97204, Germany, or Ferraris KOKO, Louisville, CO 80027, USA) according to the American Thoracic Society (ATS/ERS) guidelines (34). Forced expiratory volume in 1 second (FEV1) and forced vital capacity (FVC) values were also expressed as percentages of the predicted reference values (35, 36). Total lung capacity (TLC) was assessed through the nitrogen washout technique to measure lung volume, including TLC, inspiratory capacity (IC), and residual volume (RV). The capacity of the lungs to diffuse carbon monoxide (DLCO) and the alveolar volume (VA) were measured via a single-breath maneuver (36).

Body composition was assessed through anthropometry and bioelectrical impedance analysis (InBody 720, Teprel Medical Equipment S.A., Perafita, Portugal), and weight, body mass index, lean body mass, body fat, and waist–hip ratio were estimated (37). Ultrasonography of the diaphragmatic and appendicular muscles was performed via the LOGIQ™ system (GE Healthcare, Boston, USA). For diaphragmatic muscle assessment, a high-frequency linear transducer (10–20 MHz) was used in the intercostal region or diaphragmatic apposition zone. Diaphragm thickness at the dome was measured with either a low-frequency sector transducer (1–5 MHz) or a convex transducer (2–8 MHz), depending on the patient's body type (38). Appendicular muscle evaluation included the biceps brachii, pectoralis major, and quadriceps femoris muscles, and a high-frequency transducer (10–20 MHz) in B-mode was used to assess muscle thickness and echogenicity. The muscle echogenicity of both the appendicular and diaphragmatic muscles was analyzed via ImageJ™ software (National Institutes of Health, USA) by delineating the area with the highest muscle density to measure the average pixel histogram of the selected muscle site (39, 40).

The six-minute walk test (6MWT) was conducted according to the American Thoracic Society guidelines (41). Peripheral muscle strength was assessed in the dominant upper limb via a manual hydraulic dynamometer (SH 5001, SAEHAN), following the recommendations of the American Society of Hand Therapists (42). Respiratory muscle strength was measured by maximal inspiratory pressure (MIP) and maximal expiratory pressure (MEP) using reference values for the Brazilian population (43, 44).

The laboratory tests included complete blood count; urea, creatinine, and C-reactive protein (CRP) levels; and the levels of electrolytes (sodium, potassium, magnesium, calcium, and chloride), total proteins and fractions, aspartate aminotransferase (AST) and alanine aminotransferase (ALT).

### 2.3 Statistical analysis

The baseline was defined as the randomization visit (D1), and the end of the study was defined as the final visit (EoT). Descriptive statistics were used to characterize the participants. Analyses were conducted according to the intention-to-treat principle. Differences-in-differences (DiD) between baseline and the end of the study were estimated using general linear models with Gaussian errors and identity link and compared among the creatine supplementation groups and the control group. Isolated within-group pre–post comparisons were not independently tested, as the difference-in-differences framework was prespecified to estimate relative treatment effects compared with the control group. Between-group effects were assessed through model-based estimates and corresponding *p*-values. Categorical variables were compared using Pearson's chi-square test. A two-sided significance level of 5% was adopted. All analyses were performed via Jamovi version 2.7.12 (The Jamovi Project, Sydney, Australia) and SPSS version 20.0 (IBM, Armonk, NY, USA).

## 3 Results

**Figure 1:**
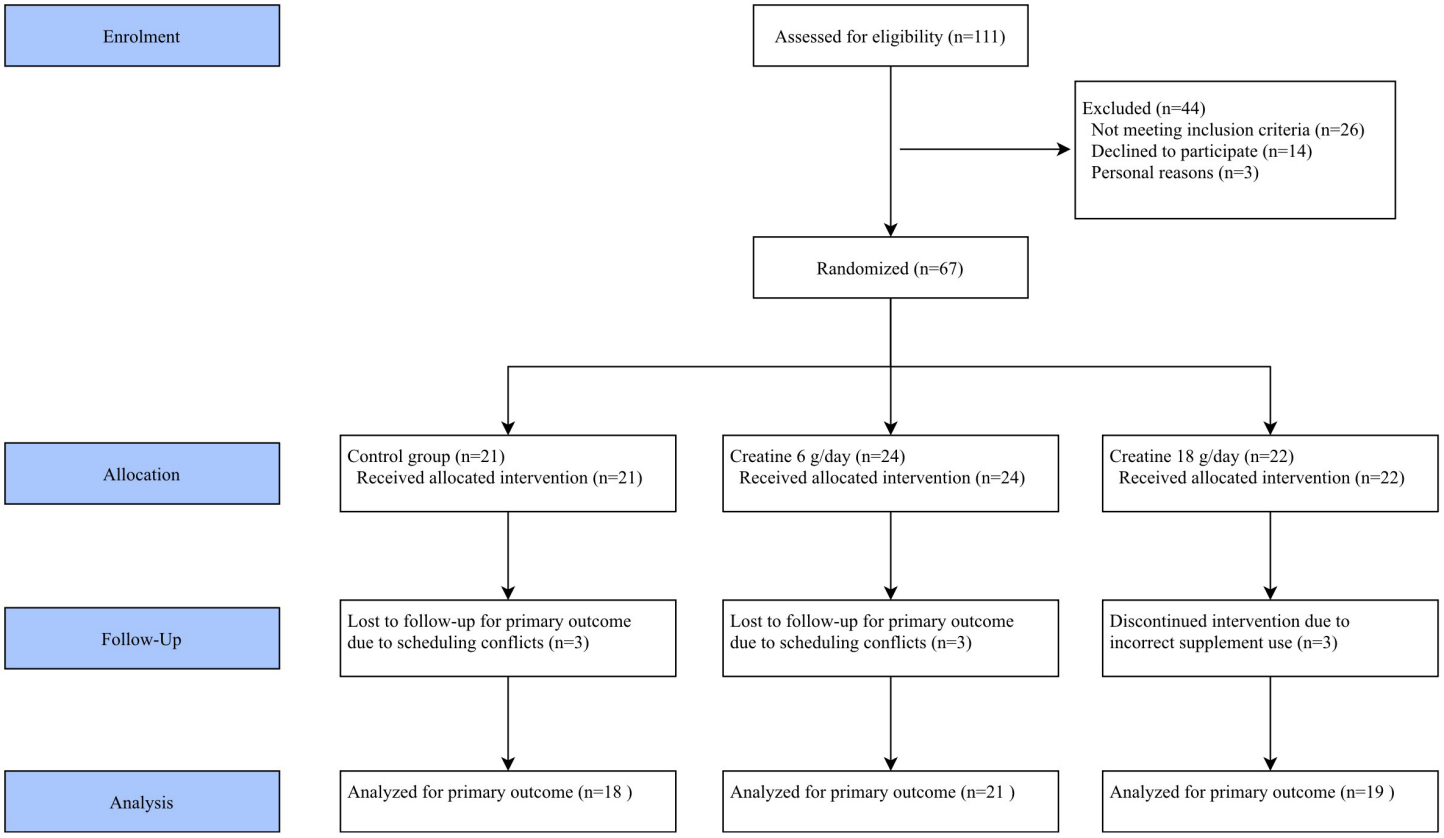
Flow diagram.

Between October 2023 and September 2024, 67 participants were enrolled, with 58 individuals completing the protocol (74% women, aged 52 ± 12 years). The intervention and comparator were delivered as planned, with high participant adherence and full fidelity to the protocol (Figure 1). No differences were found between the groups at baseline (Table 1). In contrast, a significant reduction in the PFS-R score was observed in the group receiving 6 g of creatine per day, with a mean difference (DiD estimate) of −2.05 [95% confidence interval (CI): −3.47 to −0.63; *p* = 0.005] (Table 2).

**Table 1:** General baseline characteristics of the sample.

**Variables**	**Control group *N* = 21**	**Creatine 6 g/day *N* = 24**	**Creatine 18 g/day *N* = 22**	***p*-value**
Female sex, *n* (%)	15 (71.4)	17 (70.8)	17 (77.3)	0.866
Age, years, mean (SD)	48.6 (10.8)	54.6 (12.5)	52.9 (13.0)	0.217
Race/ethnicity, *n* (%) White Mixed-race Black Asian	20 (95.2) 1 (4.8) – –	24 (100) – – –	19 (86.4) – 2 (9.1) 1 (4.5)	0.200
Body composition Weight, kg, median (IQR) Height, *m*, mean (SD) BMI, kg/m^2^, median (IQR)	78.7 (71.0–92.9) 1.62 (0.07) 30.4 (28.3–35.9)	83.6 (75.7–94.9) 1.62 (0.08) 32.1 (28.6–37.2)	85.4 (71.5–93.5) 1.64 (0.08) 31.4 (27.2–35.5)	0.782 0.663 0.805
Smoking history, *n* (%)	7 (33.3)	10 (41.7)	4 (18.2)	0.223
CCI score, median (IQR)	1.0 (0.0–2.0)	1.5 (0.0–3.0)	1.5 (0.0–3.7)	0.584
COVID-19 hospitalization, *n* (%)	7 (33.3)	10 (41.7)	8 (36.4)	0.841
Intubation, *n* (%)	3 (14.3)	4 (16.7)	4 (18.2)	0.942
PFS-R score, median (IQR)	5.5 (4.7–7.0)	5.0 (4.3–6.5)	4.9 (4.1–6.5)	0.476
SGRQ % Symptoms, median (IQR) Activities, mean (SD) Impact, median (IQR) Total, mean (SD)	21.2 (15.2–46.0) 57.1 (22.4) 23.9 (10.7–33.2) 34.3 (16.4)	36.1 (25.9–56.7) 49.5 (25.3) 23.7 (10.2–31.7) 33.9 (17.3)	31.9 (23.2–54.0) 66.6 (23.5) 25.9 (16.4–48.7) 43.1 (19.2)	0.370 0.087 0.387 0.217
mMRC Dyspnea Scale score, mean (SD)	1.3 (0.7)	1.1 (0.8)	1.7 (1.0)	0.190
BDI score, mean (SD)	8.0 (2.2)	8.0 (2.6)	6.5 (2.8)	0.120
6MWT Distance, m, mean (SD) % predicted, mean (SD)	437.4 (89.8) 80.0 (16.4)	469.8 (87.1) 87.1 (15.6)	406.1 (101.3) 76.8 (18)	0.092 0.117
Handgrip strength^†^, kgf, mean (SD)	36.3 (11.8)	31.5 (11.2)	32.3 (11.6)	0.365
MIP, cmH2O, median (IQR)	−72 (−95 to −65)	−80 (−100 to −55)	−75 (−110 to −57)	0.980
MEP, cmH2O, median (IQR)	90 (60–133)	100 (80–110)	105 (70–130)	0.640
Pulmonary function pre-BD FEV1, %, mean (SD) FVC, %, mean (SD) FEV1/FVC, ratio, median (IQR) TLC, %, mean (SD) DLCO, %, mean (SD)	89.3 (13.2) 90.5 (12.5) 0.82 (0.77–0.85) 89.1 (19.0) 91.1 (12.4)	86.3 (17.0) 89.6 (16.1) 0.80 (0.75–0.83) 91.9 (20.4) 87.8 (17.3)	78.7 (18.3) 81.7 (19.0) 0.81 (0.76–0.84) 86.9 (17.7) 81.3 (23.9)	0.135 0.237 0.473 0.748 0.328
HADS score Anxiety, mean (SD) Depression, median (IQR)	9.5 (4.7) 6.0 (2.0–10.0)	7.4 (4.1) 4.5 (1.0–7.0)	7.4 (3.9) 3.5 (2.0–7.0)	0.233 0.487

BMI, Body mass index; SD, Standard deviation; IQR, Interquartile range 25–75%; CCI, Charlson Comorbidity Index; PFS-R, Revised Piper Fatigue Scale; SGRQ, St. George's Respiratory Questionnaire; mMRC, Modified Medical Research Council Dyspnea Scale; BDI, Baseline Dyspnea Index; 6MWT, Six-minute walk test; m, meters; kgf, Kilogram-force; MIP, Maximal inspiratory pressure; MEP, Maximal expiratory pressure; BD, Bronchodilator; FEV1, Forced expiratory volume in 1 second; FVC, Forced vital capacity; TLC, Total lung capacity; DLCO, Diffusing capacity of the lung for carbon monoxide; HADS, Hospital Anxiety and Depression Scale. ^†^Handgrip measured in dominant limb.

**Table 2:** DID analysis comparing treatment groups (6 g/day and 18 g/day) with the control group.

**Variables**	**6 g/day vs. control DID (95% CI)**	***p*-value 6 g/day vs. control**	**18 g/day vs. control DID (95% CI)**	***p*-value 18 g/day vs. control**
PFS-R score	−2.05 (−3.47, −0.63)	0.005^*^	0.06 (−1.41, 1.52)	0.942
Weight, kg	0.05 (−1.26, 1.37)	0.933	0.15 (−1.17, 1.48)	0.816
BMI, kg/m^2^	0.22 (−0.42, 0.85)	0.496	−0.01 (−0.65, 0.63)	0.984
FFM, kg	0.15 (−0.69, 1.00)	0.715	0.32 (−0.56, 1.19)	0.470
FM, kg	−0.19 (−1.27, 0.89)	0.732	0.15 (−0.97, 1.27)	0.787
Biceps brachii Long head R, cm Long head L, cm Short head R, cm Short head L, cm	0.11 (−0.45, 0.67) 0.20 (−0.43, 0.84) −0.03 (−0.49, 0.44) 0.40 (−0.10, 0.90)	0.688 0.526 0.914 0.117	0.40 (−0.16, 0.97) 0.15 (−0.48, 0.80) 0.42 (−0.04, 0.90) 0.52 (0.01, 1.03)	0.162 0.623 0.076 0.045^*^
Pectoralis major R, cm Pectoralis major L, cm	−0.01 (−0.27, 0.27) 0.31 (−0.16, 0.79)	0.984 0.189	−0.22 (−0.49, 0.05) 0.15 (-0.33, 0.64)	0.110 0.523
Quadriceps femoris Rectus femoris R, cm Rectus femoris L, cm Vastus medialis R, cm Vastus medialis L, cm	0.18 (−0.14, 0.50) 0.10 (−0.17, 0.38) 0.10 (−0.28, 0.50) 0.19 (−0.12, 0.51)	0.270 0.436 0.580 0.218	0.13 (−0.19, 0.46) 0.00 (−0.28, 0.28) −0.01 (−0.41, 0.38) −0.04 (−0.36, 0.27)	0.403 0.992 0.948 0.795
Diaphragm Right dome, cm Left dome, cm	−0.02 (−0.12, 0.09) −0.01 (−0.08, 0.05)	0.767 0.699	−0.04 (−0.14, 0.06) −0.04 (−0.10, 0.02)	0.445 0.207
SGRQ Symptoms, % Activities, % Impact, % Total, %	−11.09 (−26.74, 4.56) −4.72 (−23.00, 13.55) −8.80 (−19.29, 1.69) −8.38 (−20.42, 3.64)	0.161 0.605 0.098 0.167	−0.25 (−16.72, 16.22) 0.62 (−18.69, 19.95) −0.93 (−11.97, 10.10) −1.65 (−14.37, 11.06)	0.976 0.948 0.865 0.795
mMRC, score	0.07 (−0.48, 0.64)	0.788	0.10 (−0.48, 0.68)	0.733
BDI, score	0.85 (−1.25, 2.96)	0.418	0.33 (−1.84, 2.51)	0.760
6MWD, m	−19.91 (−73.89, 34.07)	0.462	15.60 (−39.71, 70.91)	0.573
Handgrip strength^†^, kgf	4.40 (0.27, 8.52)	0.037^*^	0.67 (−3.59, 4.94)	0.751
MIP, cmH2O	−11.50 (−29.34, 6.33)	0.200	−6.62 (−25.22, 11.98)	0.477
MEP, cmH2O	8.21 (−9.00, 25.42)	0.341	−14.47 (−32.19, 3.24)	0.107
Pulmonary function pre-BD FVC, % FEV1, % FEV1/FVC, ratio TLC, % DLCO, %	2.34 (−2.52, 7.21) 1.11 (−4.13, 6.35) −0.02 (−0.04, 0.01) −4.46 (−11.69, 2.78) −0.66 (−11.06, 9.72)	0.338 0.672 0.132 0.218 0.897	1.40 (-3.69, 6.49) 0.73 (−4.74, 6.21) −0.01 (−0.03, 0.02) 1.77 (−6.51, 10.06) 1.48 (−8.90, 11.87)	0.582 0.789 0.551 0.665 0.773
HADS Anxiety, score Depression, score	0.25 (−2.53, 3.03) −0.45 (−2.47, 1.57)	0.858 0.656	0.84 (−2.04, 3.73) 1.64 (−0.44, 3.73)	0.558 0.120

PFS-R, Piper Fatigue Scale—Revised; BMI, Body Mass Index; FFM, Fat-Free Mass; FM, Fat Mass; IQR, Interquartile range (25–75%); KGF, Kilogram-force; SD, Standard deviation; MIP, Maximal Inspiratory Pressure; MEP, Maximal Expiratory Pressure; 6MWD, Six-minute walk distance; m, meters; SGRQ, St George's Respiratory Questionnaire; BDI, Baseline Dyspnea Index; mMRC, Modified Medical Research Council dyspnea scale; HADS, Hospital Anxiety and Depression Scale; FVC, Forced Vital Capacity; FEV1, Forced Expiratory Volume in 1 second; TLC, Total Lung Capacity; DLCO, Diffusing Capacity of the Lung for Carbon Monoxide. ^†^Handgrip measured in dominant limb. ^*^p <0.05 vs. control.

The analysis of post-intervention differences in body composition and muscle strength outcomes between the 6 g/day and 18 g/day groups and the control group revealed significant differences in handgrip strength in the 6 g/day group, with a mean difference (DiD estimate) of 4.40 kgf (95% CI: 0.27 to 8.52; *p* = 0.037). With the exception of one isolated ultrasonographic parameter in the 18 g/day group (DiD estimate: 0.52 cm; 95% CI: 0.01 to 1.03; *p* = 0.045), no statistically significant between-group differences were observed for the other analyzed outcomes, including quality of life (SGRQ domains), anxiety and depression symptoms, dyspnea scores, 6-min walk distance, respiratory muscle strength, body composition, muscle ultrasound parameters, and pulmonary function variables (Table 2).

Among the laboratory parameters, the 6 g/day group showed significantly lower eosinophil percentage (DiD −0.66%; 95% CI – 1.21 to −0.09; *p* = 0.022) and absolute eosinophil count (DiD −0.04 × 10^3^/mm^3^; 95% CI −0.07 to −0.01; *p* = 0.031) compared with the control group. In addition, serum creatinine was significantly higher in both the 6 g/day (DiD 0.18 mg/dL; 95% CI 0.05 to 0.32; *p* = 0.007) and 18 g/day groups (DiD 0.21 mg/dL; 95% CI 0.06 to 0.35; *p* = 0.004). The 18 g/day group also showed a modest but statistically significant increase in serum sodium (DiD 2.00 mmol/L; 95% CI 0.09 to 3.92; *p* = 0.040) compared with controls (Supplementary Table 1).

The adverse effects in the control group were restricted to one case of nausea (4.7%) and one case of headache (4.7%). In the 6 g/day group, gastrointestinal events predominated, with a higher frequency of nausea (22.7%), followed by abdominal distension (4.5%), diarrhea (4.5%), gastralgia (4.5%), flatulence (4.5%), and constipation (4.5%). In the 18 g/day group, a greater number of events were observed, notably nausea (42.8%), abdominal distension (19%), diarrhea (14%), and gastralgia (4.7%). The comparison between groups revealed no statistically significant difference (*p* > 0.05). All adverse events tended to resolve within the first few days without requiring medical intervention.

## 4 Discussion

This pilot study suggested that creatine may safely reduce fatigue symptoms and increase peripheral muscle strength in patients with PCC. Under other conditions, supplementation improved muscle function and fatigue, and although the exact mechanisms still need to be better elucidated, its effects may be related to increased intramuscular phosphocreatine (PCr) content (19, 21, 22).

It is known that creatine increases PCr availability, which is fundamental for rapid ATP resynthesis during high-intensity activities, contributing to better muscle performance. This rapid resynthesis is possible owing to the system known as the phosphocreatine shuttle, in which phosphocreatine transports high-energy phosphates from inside the mitochondria to muscle contraction sites, ensuring an efficient energy supply during exercise (45).

In the PCC, recent studies indicate that mitochondrial dysfunction is one of the central pathophysiological mechanisms associated with reduced ATP production and, consequently, manifestations of symptoms such as chronic fatigue. Evidence indicates that SARS-CoV-2 infection can cause persistent damage to mitochondria in various organs beyond the lungs, promoting systemic alterations in cellular energy generation and increased oxidative stress (46). At the molecular level, viruses interfere with mitochondrial gene expression, impair oxidative phosphorylation, and induce the activation of compensatory metabolic pathways, such as glycolysis, which is less efficient in meeting the cellular energy demand (46, 47). Furthermore, this dysfunction compromises the regulation of the innate immune response, favoring the maintenance of a persistent inflammatory state that contributes to symptom chronicity (46).

In this context, experimental models revealed that creatine could also improve mitochondrial biogenesis and respiratory efficiency and protect them from oxidative damage, maintaining their functional integrity and contributing to improved muscle performance, reduced fatigue, and preservation of cellular function under normal and pathological conditions (48–50).

Moreover, studies suggest that its supplementation elevates muscle PCr levels during the transition from anaerobic to aerobic metabolism in activities requiring greater effort, resulting in a significant delay in the onset of muscle fatigue. Additionally, it is capable of optimizing PCr resynthesis after exercise, especially in middle-aged individuals (20, 22). Another proposed mechanism is its potential to increase the intracellular water content, promoting a hydration state that benefits muscle function and metabolic control, with consequent muscle strength gain, improved physical capacity, and a reduction in symptoms such as fatigue (51).

More specifically, Ranisavljev et al. (52) reported that the total creatine concentration in the brain and skeletal muscle of patients with PCC was significantly lower than the reference values established for the general population. Consistent with these findings, Slankamenac et al. suggested that its supplementation could significantly increase the levels of this compound in various brain regions, which could be related to improved neurological function and, consequently, a reduction in symptoms reported by these patients, such as body pain, respiratory problems, concentration difficulties, and general malaise. In their specific study, glucose addition potentiated this effect, facilitating a broader increase in creatine in different brain regions (23). In another study conducted by the same authors, in which creatine supplementation was evaluated for 6 months in patients with PCC, the main hypotheses for symptom improvement, beyond increased PCr, ATP resynthesis during recovery, and improved brain bioenergetics, were anti-inflammatory action, antioxidant effects, and neuromuscular modulation (24, 53, 54).

With respect to handgrip strength, we observed an increase in the 6 g/day group, and although it is not a unique marker of muscle capacity, it is known to be strongly correlated with functionality and quality of life, particularly in the elderly population (55). In the context of PCC, decreased handgrip strength has been associated with a greater risk of morbidity and mortality in this population, suggesting that strategies for strength gain in these individuals should be encouraged (56). The role of creatine in muscle strength gain is well established, as resistance training is effective for increasing muscle strength in different populations, including young adults and elderly individuals (57, 58).

Although a 1-month intervention may be insufficient to detect significant changes in some functional outcomes, functional measures commonly used in studies involving post-COVID-19 condition, such as peripheral and respiratory muscle strength, physical capacity, and body composition, are widely validated, and functional impairments as well as longitudinal changes in physical function have been consistently reported in this population (59–62).

In the same group that received 6 g/day, a slight reduction in the eosinophil percentage and absolute eosinophil count was observed throughout the follow-up period. Although statistically significant, this variation was considered without clinical relevance and was not accompanied by signs or symptoms suggestive of immune hyperreactivity, allergic reactions, or other relevant hematological alterations, reflecting more physiological variations or transient immune system adaptations than necessarily an adverse effect of the intervention. In addition, a slight increase in serum creatinine was observed in this group. Similarly, in the group that received 18 g/day, slight increases in serum creatinine and sodium were observed. These variations, despite reaching statistical significance, were also considered without clinical relevance and were not associated with clinical signs or symptoms suggestive of renal dysfunction, electrolyte imbalance, or other relevant laboratory alterations.

With respect to other outcomes, we recognize that major alterations in these parameters are not expected in just 1 month of follow-up. However, even though statistically nonsignificant, the observed variations, especially in physical capacity and body composition indicators, suggest that future investigations with longer follow-up periods should be encouraged.

Overall, the intervention was well tolerated, presenting a consistent safety profile throughout the 1-month follow-up, without evidence of relevant adverse effects or deterioration of health status. The reported side effects manifested predominantly in the first days after supplementation initiation and regressed spontaneously, without the need for medical intervention. This behavior is compatible with what is known about creatine supplementation, particularly when it is administered at relatively high doses (10–20 g/day), in which incomplete absorption can lead to compound accumulation in the intestinal lumen, increasing osmolarity and triggering diarrhea or abdominal distension. Such manifestations tend to cease within a few days, as gastrointestinal tract adaptation occurs and the absorption rate improves (63–65).

The present study has several limitations that need to be considered. The reduced sample size may have limited the statistical power to detect additional differences between groups, and the minimum period of 4 weeks may not have been sufficient to capture all potential effects of creatine supplementation in this population, in addition to the lack of detailed investigation of participants' regular dietary intake. Furthermore, although the randomized controlled design supports causal inference, the limited sample size and short follow-up period restrict the precision and generalizability of the findings. Therefore, this study aimed to preliminarily evaluate the potential benefits of creatine supplementation in this clinical context, with more detailed elucidation of the underlying physiological mechanisms in this population being the subject of future investigations. Additionally, baseline HADS scores were predominantly within the normal or borderline range, which may have limited the detection of intervention-related changes in anxiety and depression (floor effect). Given the exploratory nature of secondary outcomes and the number of comparisons performed, findings beyond the primary outcome should be interpreted cautiously and confirmed in adequately powered trials.

Notably, although intention-to-treat analysis is methodologically appropriate, its interpretation may be limited in contexts of low adherence to supplementation or exercise protocols. In the present study, this condition may have influenced the primary outcome, considering that only the group that received 6 g/day creatine experienced fatigue improvement, whereas the group that received 18 g/day creatine did not demonstrate such improvement. Additionally, it is possible that other factors contributed to these results, including a potential nonlinear dose–response relationship, in which higher doses would not provide additional benefits and could even prove counterproductive (66). Other factors may include metabolic overload, effects on cellular hydration, interactions with exercise, effects on inflammation, and even individual dose–response. An additional factor that may help explain the absence of greater benefits in the 18 g/day group is the use of a fixed-dose supplementation strategy. This approach does not account for interindividual differences in body weight and may have resulted in heterogeneous relative dosing across participants (64, 67–69). Regarding the isolated ultrasonographic finding observed in the 18 g/day group, no consistent or clinically meaningful between-group effects were observed, and this result should be interpreted cautiously given the number of comparisons performed.

In conclusion, creatine supplementation at a dosage of 6 g/day may contribute to reducing fatigue and increasing peripheral muscle strength in patients with PCC, with good tolerability and favorable safety profiles, reinforcing the biological plausibility and therapeutic potential of creatine in this population.

## Data Availability

The raw data supporting the conclusions of this article will be made available by the authors, without undue reservation.
