# Supplementary material for: Creatine supplementation on fatigue related to post-COVID-19 condition—fatigue study: a randomized controlled trial
Source: Front Nutr. 2026 Mar 25;13:1731306. doi: 10.3389/fnut.2026.1731306 (PMC13058949; doi:10.3389/fnut.2026.1731306)
Supplement: Supplementary file 1 [file Table_1.docx]

**Supplementary Table 1.** DiD analysis comparing the 6 g/day and 18 g/day groups with the control group for laboratory parameters

| Variables | 6 g/day vs Control  DID (95% CI) | p-value  6 g/day vs Control | 18 g/day vs Control  DID (95% CI) | p-value 18 g/day vs Control |
| --- | --- | --- | --- | --- |
| Hemoglobin, g/dL | -0.36 (-0.92, 0.21) | 0.212 | -0.51 (-1.10, 0.07) | 0.08 |
| Hematocrit, % | -0.45 (-2.21, 1.30) | 0.607 | -1.38 (-3.20, 0.43) | 0.133 |
| Leukocytes, ×10³/mm³ | 0.60 (-0.52, 1.73) | 0.289 | 0.07 (-1.10, 1.24) | 0.905 |
| Neutrophils, % | 0.02 (-3.68, 3.73) | 0.988 | -0.78 (-4.62, 3.05) | 0.684 |
| Neutrophils, ×10³/mm³ | 0.41 (-0.52, 1.36) | 0.381 | -0.40 (-1.37, 0.55) | 0.402 |
| Lymphocytes, % | 1.49 (-1.83, 4.81) | 0.372 | 2.02 (-1.41, 5.45) | 0.244 |
| Lymphocytes, ×10³/mm³ | 0.33 (-0.01, 0.67) | 0.057 | 0.22 (-0.12, 0.57) | 0.205 |
| Eosinophils, % | -0.66 (-1.21, -0.09) | 0.022 | -0.50 (-1.08, 0.07) | 0.086 |
| Eosinophils, ×10³/mm³ | -0.04 (-0.07, -0.01) | 0.031 | -0.03 (-0.07, 0.01) | 0.063 |
| Platelets, ×10³/mm³ | -12.08 (-31.65, 7.47) | 0.221 | -7.93 (-28.19, 12.32) | 0.436 |
| CRP, mg/dL | 0.01 (-0.31, 0.34) | 0.928 | -0.10 (-0.45, 0.24) | 0.558 |
| AST, U/L | 1.76 (-4.41, 7.94) | 0.569 | 1.04 (-5.44, 7.53) | 0.747 |
| ALT, U/L | 0.30 (-7.61, 8.22) | 0.939 | 0.28 (-8.02, 8.59) | 0.945 |
| Total protein, g/dL | 0.10 (-0.19, 0.39) | 0.492 | 0.05 (-0.24, 0.36) | 0.697 |
| Albumin, g/dL | 0.09 (-0.08, 0.27) | 0.290 | -0.03 (-0.22, 0.15) | 0.734 |
| Urea, mg/dL | 0.61 (-5.45, 6.69) | 0.839 | 1.23 (-5.14, 7.61) | 0.699 |
| Creatinine, mg/dL | 0.18 (0.05, 0.32) | 0.007 | 0.21 (0.06, 0.35) | 0.004 |
| Sodium, mmol/L | -1.01 (-2.81, 0.79) | 0.265 | 2.00 (0.09, 3.92) | 0.040 |
| Potassium, mmol/L | 0.05 (-0.17, 0.29) | 0.626 | -0.10 (-0.34, 0.14) | 0.417 |
| Magnesium, mmol/L | 0.01 (-0.09, 0.11) | 0.833 | -0.07 (-0.18, 0.03) | 0.204 |
| Calcium, mmol/L | 0.02 (-0.24, 0.29) | 0.845 | 0.23 (-0.04, 0.51) | 0.104 |
| Chloride, mmol/L | 10.69 (-11.19, 32.59) | 0.331 | 13.05 (-9.93, 36.04) | 0.259 |
| SD: standard deviation; IQR: interquartile range; CRP: C-reactive protein; AST: aspartate aminotransferase; ALT: alanine aminotransferase. *p < 0.05 vs control. | | | | |
